# Aerobic Exercise Intervention Alters Executive Function and White Matter Integrity in Deaf Children: A Randomized Controlled Study

**DOI:** 10.1155/2018/3735208

**Published:** 2018-04-30

**Authors:** Xuan Xiong, Li-Na Zhu, Xiao-xiao Dong, Wei Wang, Jun Yan, Ai-Guo Chen

**Affiliations:** ^1^College of Physical Education, Yangzhou University, Yangzhou, Jiangsu 225127, China; ^2^Department of Medical Imaging, The Affiliated Hospital of Yangzhou University, Yangzhou University, Yangzhou, Jiangsu 225009, China

## Abstract

This study examined the effects of an 11-week aerobic exercise intervention on executive function (EF) and white matter integrity (WMI). In total, 28 deaf children (aged 9–13 years) were randomly assigned to either an 11-week exercise intervention or the control group. All the children had behavioral assessment and diffusion tensor imaging prior to and following the exercise intervention. The behavioral performance results demonstrated that EF was enhanced by exercise. Relative to the control group, WMI of the exercise intervention group showed (1) lower fractional anisotropy (FA) in the pontine crossing tract (PCT) and right cingulum (hippocampus) (CH), genu of the corpus callosum (gCC), right inferior cerebellar peduncle (ICP), left superior corona radiata (SCR), and left superior frontooccipital fasciculus (SFOF); (2) higher mean diffusivity (MD) in the gCC, right CH, right inferior frontooccipital fasciculus (IFOF), and left anterior limb of the internal capsule (ALIC); and (3) lower MD in the left ICP and left tapetum (TAP). Furthermore, the lower FA in gCC showed a significant negative correlation with improvement in behavioral performance, but the correlation was not significant after FDR correction. These results suggest that exercise can effectively improve deaf children's EF and reshape the WMI in deaf children. The improved EF by exercise is not related to a reshaping of WMI, but more studies on the relationship between EF and WMI by exercise may be needed.

## 1. Introduction

Executive function (EF), including inhibition, working memory, and shifting, refers to higher and meta-levels of cognitive processes that regulate and organize purposeful and goal-directed behaviors [1, 2] and is at the core of children's cognition, emotion, and social function, playing an important role in the development of children's mental health [3, 4]. Deficits in EF will seriously harm the development of children's physical, mental, and social achievements; conversely, individuals, local communities, and society will benefit from well-developed EF [5–7]. EF is based on the dynamic interaction between the prefrontal cortex and other cortical and subcortical regions [8], and it is flexible and plastic and can therefore be improved through training, especially in high correlation with children's cognitive development [9, 10]. Various fields have paid attention to EF—particularly at the frontier of interdisciplinary research—as the key to effective methods for improving children's EF.

A burgeoning body of literature has emerged on the positive effects of aerobic exercise on the brain and EF. Exercise plays a causal role in improving EF, as exercise training improves performance of EF tasks [11]. A 10-week aerobic exercise program in primary students with Chinese learning difficulties improved EF performance in the exercise group compared to the control group [12]. Another study of deaf children found positive effects on working memory and shifting of executive function in preadolescent deaf children after an 8-week moderate skipping training program [13]. Nevertheless, it remains unclear whether the neural basis of improvement in deaf children's EF is elicited by exercise intervention.

Exercise intervention improved EF and altered brain activation as assessed by functional magnetic resonance imaging (fMRI). Specifically, a 6-month exercise intervention in older adults improved performance and increased prefrontal and posterior parietal activation during a flanker task in the exercise group as compared to the controls [14]. Changes in regions of the brain were also found in studies of children. Our group recently found that 11-week exercise intervention in children aged between 9 years and 13 years improved EF performance compared to the controls. The exercise group also increased frontal lobe, temporal lobe, hippocampus, and cingulate cortex activation during an EF task compared to the control group [15].

With evidence that brain activation is affected by exercise, one issue that warranted investigation was whether exercise alters brain structure. Altered white matter structure may be an underlying cause of functional change, given the evidence that interindividual differences in brain activation reflect white matter integrity (WMI) [16]. WMI reflects axonal membrane structure and myelination and can be assessed by diffusion tensor imaging (DTI), which measures the anisotropy (directional dependence) of water diffusion. Fractional anisotropy (FA) is a frequent measure of interest in DTI and describes the anisotropy of water diffusion. FA values range between 0 and 1, with 1 indicating fully anisotropic diffusion. Higher values are generally interpreted as greater WMI (myelination and axonal membrane structure [17]). Another measure based on the same tensor model is mean diffusivity (MD), which measures water diffusion restricted by water (with higher values indicating less restriction). Taken together, higher FA and lower MD values are often interpreted as primarily reflecting greater myelination.

WMI has been associated with fitness in several cross-sectional studies. Higher aerobic fitness in adults was associated with higher FA in the cingulum and corpus callosum, possibly relating to motor planning and control [18–20]. Fitness was also associated with the integrity of the uncinate fasciculus, which is involved in memory [20]. The longitudinal study also found that the improvement in the WMI of overweight children after an 8-month exercise intervention was related to selective attention [21]. While the literature indicates that exercise affects many cognitive abilities and WMI, this topic has yet to be investigated in relation to EF.

Given the evidence that exercise improved EF and altered associated brain activation in prior studies, we investigated whether an exercise intervention in deaf children improves WMI. Only deaf children were recruited for the current study; the EF of deaf children is retarded, and they are therefore likely to derive greater benefits from exercise [22]. As the brain structure does not completely mature until young adulthood, ongoing development makes it an interesting target for investigation across the ages included in the current study (children aged between 9 years and 13 years). Our hypotheses were generated based on the literature indicating that exercise improves both EF and WMI. Specifically, we hypothesized that a randomized controlled exercise intervention with deaf children would improve their EF behavioral performance and reshape their WMI. Further, improved EF in deaf children may be associated with WMI changes after an exercise intervention, which may help us better understand the biological mechanisms underlying these changes.

## 2. Materials and Methods

### 2.1. Participants

The 28 deaf children recruited from two special education schools who participated in the study had normal or corrected-to-normal vision and were right-handed as assessed by the Edinburgh Test [23]. All participants were free of psychiatric disorders or a history of head trauma. They also completed a set of questions relating to their history of drug abuse or inherited disease and their general intelligence. Exclusions included any medical condition that would limit exercise intervention or affect study results (including neurological or psychiatric disorders). The study was conducted in accordance with the Declaration of Helsinki.

All participants were then randomly assigned to either the control or the exercise intervention group. The exercise group included six females and eight males. The other six females and eight males constituted the control group. Age and gender were well matched between the two groups. MRI was completed with DTI data available for 28 children at baseline and 20 at posttest. Of the 20 children with both baseline and posttest data, one was excluded due to the loss of behavioral performance data and the other was excluded because behavioral performance was an extreme outlier. Thus, the present study included 18 children: 10 in the exercise group and 8 in the control group (Table 1). The study protocol was approved by the Ethics and Human Protection Committee of the Affiliated Hospital of Yangzhou University. Written informed consent was obtained from each participant after the experimental procedures had been fully explained.

### 2.2. Exercise Intervention

The aerobic exercise program was adapted from Chen et al. [24] and Yin et al. [25]. All subjects in the exercise group were offered an after-school program 4 days per week for 11 weeks. The exercise program consists of three stages, (1) preparation, (2) exercise intervention, and (3) relaxation, all lasting for about 45 minutes. The first stage consisted of warm-up exercises (preparation stage), wherein exercise intensity reached a moderately intense heart rate. This was followed by a 30 min exercise stage that emphasized exercise intensity, enjoyment, safety, repetition, and practice; all activities were selected based on the ease of comprehension, fun, and eliciting of intermittent vigorous movement, including running games, jumping rope, and wushu. The chosen aerobic exercise load was of moderate intensity [60%–69% of maximum heart rate (MHR), wherein MHR = 220—age], based on the aerobic exercise intensity classification defined by the American College of Sports Medicine [26]. Exercise intensity was monitored by heart rate monitors (Polar Electro RS800XSD, Oy, Finland) that were attached throughout the experiment to four subjects (two boys and two girls). The final (relaxation) stage focused on physically relaxing prior to ending the exercise regime.

### 2.3. Executive Function and Related Assessments

A test-tool designed by Chen et al. [11] was used to assess EF of deaf children. Three computer-based neuropsychological assessments were used to assess inhibition, working memory, and shifting aspects of EF. A modified Eriksen flanker task was used to examine the inhibitory control aspect of EF [27]; the response times (RT) in the congruent and incongruent trials were recorded and used to create an index of inhibition, defined as the RT difference between incongruent and congruent trials. Shorter RT differences reflected better performance. A 2-back task was used to assess the working memory aspect of EF. The RT on correct trials were recorded and averaged as the main behavioral index, wherein shorter RT reflected better performance. A more-odd task adapted from Hillman et al. [28] and Salthouse et al. [29] was employed to investigate the shifting aspect of EF. The shifting index used in the present study was the global switch cost, which was calculated as the RT difference between the heterogeneous (i.e., the average of the c blocks) and homogeneous (i.e., the average of the a and b blocks) blocks. The detail of every test was introduced in our previous paper [11]. The stimulus presentation and response data collection was performed using E-Prime software 1.1 (Psychology Software Tools Inc., Pittsburgh, USA).

### 2.4. DTI Procedure and Analysis

#### 2.4.1. MRI Acquisition

Images were acquired at the Affiliated Hospital of Yangzhou University on a Siemens Magnetom Tim Verio 3 Tesla scanner. During scanning, head position was stabilized with a vacuum pillow and/or foam padding. Diffusion images were acquired using an echo planar imaging sequence (acquisition matrix = 128 × 128, 60 interleaved slices, voxel size = 1.8 × 1.8 × 4.0 mm, FOV = 230 × 230 mm, TR = 3800 ms, TE = 106 ms, 3 B0 images, 30 diffusion-weighted images, and b = 1000 s/mm^2^).

#### 2.4.2. Image Analysis

Diffusion images were processed using a MATLAB toolbox named “Pipeline for Analyzing braiN Diffusion imAges (PANDA) (http://www.nitrc.org/projects/panda/)” [30]. The main procedures included preprocessing and producing diffusion metrics in preparation for statistical analysis: local diffusion homogeneity (LDH) = 7 voxels, smooth: normalizing resolution = 2 mm, and smoothing kernel = 6 mm. The preprocess steps were executed one by one, including converting DICOM files into Nifti images, estimating the brain mask, cropping raw images, correcting for the eddy-current effects, and calculating diffusion tensor metrics. Then, using the atlas-based analysis, we normalized diffusion metrics (FA and MD) into the MNI space and calculated regional diffusion metrics by averaging the values within each region of the ICBM DTI-81 atlas [31]. All the procedures were fully automated and completed by PANDA.

### 2.5. Experimental Process

**Figure 1:**
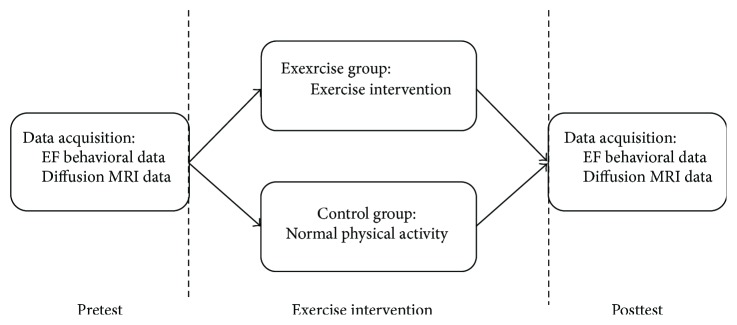
Experimental process.

In this longitudinal study, participants were scanned twice with MRI. MRI was performed as follows: a pretest scan performed before exercise intervention (MRI 1) and a posttest scan 11 weeks after completion of the intervention period (MRI 2). The control group, consisting of age- and gender-matched subjects scanned at pretest and posttest, did not participate in any additional aerobic exercise during the 11 weeks (Figure 1).

### 2.6. Statistical Analyses

All analyses were conducted using SPSS Version 20.0 (IBM, Armonk, N.Y., USA). Demographic variables were compared between the control and exercise groups with independent sample *t*-tests for continuous variables and *χ*^2^ tests for sex proportion. For the performance of executive function tasks, group-by-time repeated-measures analyses of variance (ANOVAs) were conducted separately for inhibition, working memory, and shifting indices, whereas effect size was presented as partial eta-squared (*η*^2^) values. Post hoc analyses were conducted with planned pairwise comparisons when significant interaction effects were revealed. Repeated measures ANOVAs were conducted for both measurements of WMI (FA and MD) on the outcome to examine the effects of group, measurement time, and their interactions. Each model was also controlled for age. Then, we calculated the Pearson correlation coefficient (*r*) between WMI and EF performance in the exercise group. Results were corrected for multiple comparisons using the false discovery rate (FDR) correction. Probability values < 0.05 were considered statistically significant.

## 3. Results

### 3.1. Participants' Characteristics

The participants' demographic details are presented in Table 1. Independent *t*-tests revealed no significant differences between the control and exercise groups in terms of gender (chi − square = 0.18, *P* > 0.05) or BMI [*t* (16) = 0.027, *P* > 0.05], but because of the reduction in data, the age of the two groups was inhomogeneous; therefore, we used “age” as a covariate in the subsequent statistical analysis to eliminate its influence.

### 3.2. Exercise Intensity Manipulation

The heart rates for the control and exercise groups were 42.52% and 64.95% of the maximal heart rate, respectively [*t* (6) = 9.13, *P* < 0.05]. The different heart rates between the two treatment groups, as well as the percentages of the maximal heart rates, suggested that the consideration of the exercise manipulation of moderate exercise intensity was appropriate.

### 3.3. Behavioral Performance

The groups did not differ significantly at baseline on any of the characteristics listed in Table 2. Based on a priori hypotheses concerning the effects of physical exercise on cognition, the two groups of deaf children were compared with an ANOVA. A repeated measures ANOVA was conducted to examine group differences in behavioral performance, with time (pretest and posttest) as the within-subject factor and group (exercise intervention and control) as the between-subject factor.

#### 3.3.1. Inhibition

A repeated measures ANOVA revealed the main effects to be time [*F*(1, 16) = 1.61, *P* > 0.05, partial *η*^2^ = 0.09] and group [*F*(1, 16) = 0.04, *P* > 0.05, partial *η*^2^ = 0.003]. There was no significant difference between both of them. The interaction between time and group was significant [*F*(1, 16) = 9.05, *P* < 0.01, partial *η*^2^ = 0.38]. A follow-up analysis deconstructing the interaction revealed significant pretest [*F*(1, 16) = 5.68, *P* < 0.05] and posttest [*F*(1, 16) = 8.20, *P* < 0.05] differences between the exercise and control groups (i.e., the RT of the exercise group were lower than those of the control group after exercise); there was no significant difference between pre- and posttest results in the control group [*F*(1, 16) = 2.84, *P* > 0.05], but there was a significant difference in pre- and posttest condition in the exercise group [*F*(1, 16) = 20.66, *P* < 0.001]; the posttest inhibition RT differences was shorter than the pretest inhibition RT differences in the exercise group.

Regarding accuracy, no significant interaction effect was observed in inhibition effect regarding “congruent” [*F*(1, 16) = 0.34, *P* > 0.05, partial *η*^2^ = 0.02] and “incongruent” [*F*(1, 16) = 0.34, *P* > 0.05, partial *η*^2^ = 0.02] behaviors.

#### 3.3.2. Working Memory

A repeated measures ANOVA revealed the main effects for time [*F*(1, 16) = 4.38, *P* > 0.05, partial *η*^2^ = 0.23] to be not significant, but those for group [*F*(1, 16) = 6.51, *P* < 0.05, partial *η*^2^ = 0.30] were significant. The interaction of time and group was significant [*F*(1, 16) = 18.63, *P* < 0.05, partial *η*^2^ = 0.55]. A follow-up analysis deconstructing the interaction effects revealed no significant pretest differences in RT between the exercise and control groups [*F*(1, 16) = 1.99, *P* > 0.05] or in posttest differences [*F*(1, 16) = 0.15, *P* > 0.05]. (However, the RT of the exercise group were longer than those of the control group after exercise.) There was no significant difference between pre- and posttest RT in the control group [*F*(1, 16) = 1.68, *P* > 0.05], but there was a significant difference in the pre- and posttest RT in the exercise group [*F*(1, 16) = 34.35, *P* < 0.001] (i.e., in the exercise group, the posttest RT were less than the pretest RT).

Regarding accuracy, no significant interaction effect was observed in working memory [*F*(1, 16) = 0.06, *P* > 0.05, partial *η*^2^ = 0.004].

#### 3.3.3. Shifting

A repeated measures ANOVA revealed the main effects to be time [*F*(1, 16) = 0.01, *P* > 0.05, partial *η*^2^ = 0.001] and group [*F*(1, 16) = 0.44, *P* > 0.05, partial *η*^2^ = 0.03]. There was no significant difference between both of them. The interaction between time and group was significant [*F*(1, 16) = 5.07, *P* < 0.05, partial *η*^2^ = 0.25]. A follow-up analysis deconstructing the interaction effects revealed no significant differences in pretest [*F*(1, 16) = 0.00, *P* > 0.05] or posttest [*F*(1, 16) = 4.33, *P* > 0.05] conditions between the exercise and control groups (however, the RT of the exercise group were lower than those of the control group after exercise intervention); there was no significant difference in RT between pre- and posttest conditions in the control group [*F*(1, 16) = 4.35, *P* > 0.05] or exercise group [*F*(1, 16) = 3.89, *P* > 0.05]. (However, for the exercise group, the posttest shift in RT was shorter than the pretest shift in RT.)

Regarding accuracy, no significant interaction for shifting was observed in “homogeneous” [*F*(1, 16) = 1.01, *P* > 0.05, partial *η*^2^ = 0.06] or “heterogeneous” [*F*(1, 16) = 3.67, *P* > 0.05, partial *η*^2^ = 0.19].

### 3.4. WM Structure

**Figure 2:**
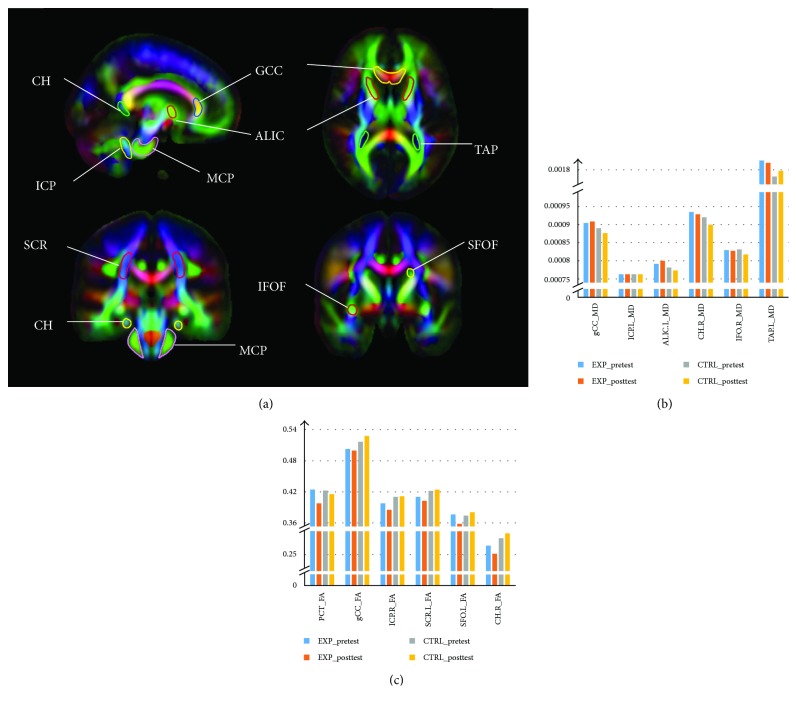
(a) nine anatomical regions defined by the ICBM DTI-81 atlas with significant changes after exercise intervention. Difference in MD (b) and FA (c) values for specific fiber tracts in an atlas-based ROI analysis between the experimental group (blue, orange) and the control group (gray, yellow).

There was a significant group-by-time interaction between groups in some WMI measures (Figure 2): a decreased FA in the pontine crossing tract (PCT) [*F*(1, 16) = 6.83, *P* < 0.05, partial *η*^2^ = 0.31] and right cingulum (hippocampus) (CH) [*F*(1, 16) = 8.96, *P* < 0.01, partial *η*^2^ = 0.37], genu of the corpus callosum (gCC) [*F*(1, 16) = 6.20, *P* < 0.05, partial *η*^2^ = 0.29] and left superior frontooccipital fasciculus (SFOF) [*F*(1, 16) = 5.42, *P* < 0.05, partial *η*^2^ = 0.27], right inferior cerebellar peduncle (ICP) [*F*(1, 16) = 4.95, *P* < 0.05, partial *η*^2^ = 0.25], and left superior corona radiata (SCR) [*F*(1, 16) = 6.06, *P* < 0.05, partial *η*^2^ = 0.29]; an increased MD in the genu of the corpus callosum (gCC) [*F*(1, 16) = 7.37, *P* < 0.05, partial *η*^2^ = 0.33] and left anterior limb of the internal capsule (ALIC) [*F*(1, 16) = 4.89, *P* < 0.05, partial *η*^2^ = 0.25], right inferior frontooccipital fasciculus (IFOF) [*F*(1, 16) = 6.80, *P* < 0.05, partial *η*^2^ = 0.31], and and right cingulum (hippocampus) (CH) [*F*(1, 16) = 8.43, *P* < 0.05, partial *η*^2^ = 0.36]; and a lower MD in the left inferior cerebellar peduncle (ICP) [*F*(1, 16) = 4.66, *P* < 0.05, partial *η*^2^ = 0.24] and left tapetum (TAP) [*F*(1, 16) = 4.98, *P* < 0.05, partial *η*^2^ = 0.25]. These results indicate that the exercise intervention differentially affected WMI compared to that of the control condition.

### 3.5. Correlations between WMI and Behavioral Performance

We found a significant negative correlation between WMI and behavioral performance, wherein a decrease in WMI in the gCC (from pretest to posttest) was associated with a lessening in inhibition for deaf children in the exercise intervention group (Flanker task, pretest to posttest) and reaction time (*r* = −0.67, *P* = 0.03 < 0.05); however, there was no significant correlation after correction for multiple comparisons (FDR, *P* < 0.05).

## 4. Discussion

The current study was designed to explore the effects of aerobic exercise on EF and WMI in deaf children. Children from two similar special education schools were randomly allocated to two groups: an exercise intervention group, receiving an aerobic exercise intervention including running games, jumping rope, and wushu, and a control group that did not attend any additional aerobic exercise. We controlled for all the confounding variables. Consequently, reliable exercise gains emerged, allowing us to observe the neural basis of exercise-improved EF.

### 4.1. Behavioral Performance

A rapidly growing body of literature indicates that, from both behavioral and neuroelectric perspectives, physical exercise improves EF. As observed here, deaf children's EF performance in the exercise intervention group was better than that in the control group—in agreement with previous studies [11, 32–36]. Accordingly, the present behavioral results have again been verified: physical exercise beneficially impacts children's EF.

### 4.2. White Matter Integrity

Recently, a number of studies have focused on exploring the effects of exercise on the brain. Some evidence has indicated that exercise intervention can cause microstructural changes in the WM [21, 37, 38]. Our results support existing evidence that exercise intervention causes changes in WMI. Specifically, a decrease in WMI was found in the PCT, right CH, left SFOF, right IFOF, right ICP, left SCR, left ALIC, and gCC, whereas there was an increase in WMI in the left ICP and left TAP.

Previous research shows that, compared with normal-hearing subjects, the microstructural changes of brain white matter in deaf subjects have lower FA in their bilateral auditory [39–41]. Hribar et al. [42] also found lower AD in the left ALIC and left SCR in deaf individuals than in normal-hearing individuals, which are regions important for the transmission of sensory, motor, visual, auditory, and other information between the cerebral cortex, the brainstem, the cerebellum, and the spinal cord. Hribar et al. suggested that the lower anisotropy values found in the large network of projection fibers in deaf people may be due not only to the degradation of their auditory pathways but also to the reorganization of sensory, motor, and visual pathways as a compensation for the absence of auditory input. These WMI changes in brain regions were also observed in our study, wherein the FA declined or MD increased after a prolonged exercise regimen.

Higher MD in deaf children was observed also in the right IFOF. A decreased FA among deaf subjects has been previously reported for the right IFOF [42]. IFOF connects the occipital and frontal lobes [43]. Philippi et al. [44] found that damage associated with the right IFOF impairs recognition of facial expressions and emotions. For deaf subjects, facial expressions are important for interpreting a speaker's emotional state because they cannot hear a speaker's tone of voice; this is also critical for sign language comprehension [42]. Studies have shown that deaf subjects have a keen ability to recognize subtle differences in facial features, which may be related to their experience using sign language [45]. Most studies relate lower anisotropy values to demyelination and degradation of axons, which lead to poorer functioning [46]. However, lower anisotropy values might not always correlate with poorer functional performance, as shown by Hoeft et al., who indicated that increased FA values correlate with poorer visuospatial construction abilities [47]. The increased MD of IFOF of deaf children in our study may have required more recognition of facial expressions and emotional states during exercise, because both attributes are important for comprehending sign language.

Lower FA and higher MD occurred in the gCC in our study, suggesting that the lower FA could be attributable to myelination abnormalities. Previous research found that deaf subjects show lower AD in the gCC relative to normal-hearing subjects, which might be related to impaired motor proficiency and balance problems in people with sensorineural hearing loss [42]. However, in our study, physical exercise seemed to improve motor proficiency and enabled the formation of new connections and reinforcement and degradation of existing connections may also alter FA in the deaf. Because the anterior part of the CC contains fibers projecting into the prefrontal, premotor, and supplementary motor cortical areas [48], we speculate that exercise may promote a compensatory reorganization.

Our results found higher WMI in the left ICP and left TAP after exercising, while WMI was reduced in the PCT, right CH, right ICP, and left SFOF. However, similar results have not been observed in previous relevant studies. It is possible that our results only pertain to our study due to the small sample size of our study. However, even if this is true, our results may provide a basis for future research comprising more test subjects.

The change in diffusion anisotropy of deaf children implies an alteration in WMI, but we cannot draw conclusions from a one-sided index. In our study, we have shown where the changes are, but the interpretation of diffusion indices in deaf children requires further research.

### 4.3. Correlation between Behavioral Performance and WMI

We found a significant correlation in deaf children (following an imposed exercise regimen) between lower WMI in the gCC and better inhibition behavioral performance (declines in reaction time). Nevertheless, perhaps because our sample size was small or our intervention time was short, the correlation was not significant after FDR correction. The existing theory indicates that exercise improves cognitive functioning by improving brain plasticity (structural components, activation patterns, functional connectivity, etc.) [49, 50]. We had hoped to explore the neural basis of exercise on EF from the perspective of WMI plasticity, but our results did not support our initial hypothesis. In fact, our results did not provide any evidence for a significant correlation between changes in WMI and improvements in EF behavioral performance. Even so, this finding might provide an interesting a priori hypothesis for future studies and so this topic would still be worth exploring in future research.

## 5. Conclusions

Our results demonstrated that, after establishing an exercise regimen in deaf children, EF improved in three behavioral performance measures and declined for WMI in the PCT, right CH, left SFOF, right IFOF, right ICP, left SCR, left ALIC, and gCC. In addition, WMI increased in left ICP and left TAP after exercise. In summary, our results suggest that exercise intervention may reshape the microstructure of WM in deaf children, which may have some implications for the instruction of alternative sport programs for children with executive dysfunction.

## Figures and Tables

**Table 1:** Participants' demographics and treatment-induced heart rates (M ± SD).

Variables	Control group	Experimental group	*P* value
*N*	8	10	—
Sex (male/female)	4/4	3/7	0.52^b^
Age (years)	11.50 ± 0.76	10.20 ± 1.23	0.02^a^
BMI (height/weight^2^)	17.88 ± 1.46	17.90 ± 2.42	0.98^a^
*n* ^c^	4	4	—
Sex (male/female)	2/2	2/2	—
HR during treatment	89.30 ± 10.30	136.40 ± 0.57	0.03^a^

Values are presented as mean ± SD or percentages unless otherwise indicated. ^a^*t*-test. ^b^*χ*^2^ test. ^c^Heart rate value was presented for four participants in each group.

**Table 2:** Performance for three fundamental aspects of executive function (M ± SD).

	Control group		Experimental group	
	Pretest	Posttest	Pretest	Posttest
RT (ms)				
Inhibition	17.80 ± 20.70	29.88 ± 21.55	38.93 ± 16.97	9.80 ± 5.22
Working memory	651.30 ± 104.04	622.51 ± 93.36	724.09 ± 112.13	607.53 ± 71.53
Shifting	277.50 ± 206.72	339.87 ± 160.79	278.43 ± 50.00	225.69 ± 60.81
Accuracy (%)				
Inhibition				
Congruent	88.75 ± 12.08	88.13 ± 14.64	93.50 ± 6.55	95.20 ± 4.13
Incongruent	87.13 ± 11.45	87.13 ± 12.40	87.40 ± 13.60	91.00 ± 7.12
Working memory	64.00 ± 26.79	68.00 ± 19.24	83.80 ± 20.47	90.10 ± 8.48
Shifting				
Homogeneous	80.00 ± 11.46	73.34 ± 24.49	80.20 ± 15.83	84.50 ± 16.22
Heterogeneous	53.25 ± 17.91	46.88 ± 20.32	48.30 ± 30.39	63.30 ± 33.20
